# Divide and Conquer May Not Be the Optimal Approach to Retain the Desirable Estrogenic Attributes of the *Cyclopia* Nutraceutical Extract, SM6Met

**DOI:** 10.1371/journal.pone.0132950

**Published:** 2015-07-24

**Authors:** M. Mortimer, K. Visser, D. de Beer, E. Joubert, A. Louw

**Affiliations:** 1 Department of Biochemistry, Stellenbosch University, Stellenbosch, Western Cape, South Africa; 2 Post-Harvest and Wine Technology Division, Agricultural Research Council of South Africa Infruitec-Nietvoorbij, Stellenbosch, Western Cape, South Africa; 3 Department of Food Science, Stellenbosch University, Stellenbosch, Western Cape, South Africa; Wayne State University, UNITED STATES

## Abstract

The genus *Cyclopia*, an indigenous South African fynbos plant used to prepare honeybush tea, contains phytoestrogenic compounds. An extract from *C*. *subternata*, SM6Met, displays three desirable estrogenic attributes for future development of a phytoestrogenic nutraceutical, namely, ERα antagonism, ERβ agonism, and also antagonism of E_2_-induced breast cancer cell proliferation. Activity-guided fractionation of SM6Met was used in an attempt to isolate and identify compounds conferring the desirable estrogenic profile to SM6Met. Initial liquid-liquid fractionation of SM6Met yielded a polar fraction (PF) and a non-polar fraction (NPF), with the desirable estrogenic attributes retained in the NPF. Subsequent high performance counter-current chromatography (HPCCC) fractionation of the NPF yielded three fractions (F1-F3). Interestingly, the fractions revealed separation of the previously demonstrated positive estrogenic attributes of the NPF into separate fractions, with F1 and F2 acting as ERα antagonists, only F2 inducing antagonism of E_2_-induced breast cancer cell proliferation and only F3 retaining robust ERβ agonist activity. In terms of major polyphenols, quantitative HPLC and liquid chromatography tandem mass spectrometry (LC-MS/MS) indicated that HPCCC fractionation resulted in a divergence of polyphenolic classes, with F1 emerging as the dihydrochalcone-rich fraction and F2 as the flavanone- and benzophenone-rich fraction, while the xanthones, flavones and phenolic acids were retained in F3. F3 was re-engineered into F3R by reassembling the major polyphenols identified in the fraction. F3R could, however, not replicate the effect of F3. In conclusion, although activity-guided fractionation results suggest that retention of all the desirable estrogenic attributes of the original SM6Met in one fraction is not an attainable goal, fractionation is a useful tool to enhance specific desirable estrogenic attributes.

## Introduction

Nutraceuticals, i.e. food extract supplements with health benefits [1], have become a multi-billion dollar global industry [2]. Consumers have shown renewed interest in nutraceuticals as a consequence of increased public health consciousness [3], increasing costs of modern medicines [4], and increasing concerns regarding side effects associated with conventional therapies [5,6]. Africa is currently not seen as a competitive market for nutraceuticals, however, an emerging market is establishing itself in South Africa [7,8], thus opening the door for the development of a uniquely South African nutraceutical.

*Cyclopia* species (family Fabaceae) are endemic to the Western and Eastern Cape Provinces of South Africa [9], and have been harvested for more than a century for the production of an herbal tea known as honeybush tea, produced from ‘fermented’ (oxidized) plant material [10]. Renewed interest in *Cyclopia* in the 1990’s led to the production of green or ‘unfermented’ honeybush tea, in order to prevent breakdown of potentially health-promoting polyphenols during the high temperature oxidation process. This ‘unfermented’ plant material could serve as a source for nutraceutical extracts [11]. As consumers worldwide demonstrated an increased demand for health-promoting foods, the honeybush industry has benefitted through a growing export market [9]. Several *in vitro* and *in vivo* studies have already shown that several *Cyclopia* species have antimutagenic [12], antioxidant [13], anticancer [14–16] and also phytoestrogenic properties [10].

Women undergoing the menopausal transition have shown great interest in phytoestrogenic nutraceuticals as an alternative to conventional hormone replacement therapy (HRT) due to the disquieting side-effect profile of HRT [17–21], with breast cancer as a primary adverse outcome [19,20]. Studies have shown that phytoestrogen consumption cannot only alleviate menopausal symptoms, but may also lower the incidence of osteoporosis, cardiovascular disease, and hormone dependent cancers, such as breast cancer [22–28].

The possibility of *Cyclopia* as a phytoestrogen source was first raised in two studies by Verhoog *et al*. [29,30]. In the initial study [29] it was found that extracts from both *C*. *genistoides* and *C*. *subternata* displayed phytoestrogenic activity, and that optimal estrogenicity was generated by using methanol extracts, as opposed to aqueous extracts, from unfermented plant material. In the second study [30] a dried methanol extract (DME) of *C*. *genistoides*, P104, was shown to bind to both estrogen receptor (ER) subtypes, transactivate an estrogen response element (ERE) containing promoter reporter construct only via ERβ, and also antagonize 17β-estradiol (E_2_)-induced breast cancer cell proliferation. Subsequent screening of extracts produced from *C*. *genistoides* and *C*. *subternata* [31], resulted in the identification of SM6Met, a sequential methanol extract of a *C*. *subternata* harvesting, M6. SM6Met was estrogenically the most potent of the screened *Cyclopia* extracts, with potency comparable to commercially available phytoestrogenic nutraceuticals [31]. More recently, investigation into the estrogenic specificity of SM6Met revealed that the extract displays three desirable estrogenic attributes for future development of a phytoestrogenic nutraceutical, namely, ERα antagonism, ERβ agonism, and also antagonism of E_2_-induced breast cancer cellular proliferation [32]. This suggests that SM6Met contains a compound or compounds conferring subtype specific estrogenic activity [33], which may be beneficial as it has been shown that physiologically, ERα is associated with the stimulation of cell proliferation and the increased occurrence of breast cancer, while ERβ diminishes the effect of ERα in breast cancer and could act as an inhibitor of breast cancer development [34–41]. Thus a phytoestrogenic nutraceutical with the desirable estrogenic attributes displayed by SM6Met may alleviate menopausal symptoms through ERβ activation, whilst as a positive side effect it might prevent the development of breast cancer through ERα antagonism [42].

For the future development of a phytoestrogenic nutraceutical, the candidate formulation, as for any new nutraceutical, should meet important prerequisites for the marketing of health claims [43]. One important prerequisite is formulation standardization, which includes efficacy, quality and safety data, however, equally important prerequisites include identification of the active compound(s), and elucidation of the molecular mechanism of action, which covers amongst others absorption, distribution, metabolism and excretion (ADME) of the active compound(s) [44–47]. Identification of the compounds conferring the desirable estrogenic attributes to SM6Met is not only important for the manufacturing process of a quality-assured phytoestrogenic nutraceutical [43], but will also be important for screening prospective *Cyclopia* harvestings destined for nutraceutical production and as marker compound(s) that could in future be used to direct plant breeding programs of *Cyclopia* plants for nutraceutical production.

The current study, part of a larger study investigating the development of a phytoestrogenic nutraceutical from *Cyclopia*, focuses on activity-guided fractionation of SM6Met from *C*. *subternata* to identify the compound(s) responsible for its desirable estrogenic attributes, namely ERβ agonism, ERα antagonism and antagonism of E_2_-induced breast cancer cell proliferation [30–32]. Promoter reporter and breast cancer cell proliferation assays were employed to monitor estrogenic activity and quantitative HPLC (qHPLC) and LC-MS/MS were used to track polyphenols during fractionation for correspondence with positive estrogenic attributes. High performance counter-current chromatography (HPCCC) was selected for fractionation due to the advantage of complete recovery of the extract in contrast to liquid chromatography using a solid stationary phase. Finally, we re-engineered [48] the most promising fraction using the major polyphenols identified and comparing estrogenic activity with that of the original fraction.

## Materials and Methods

### Compounds

The following compounds were used in cell culture for estrogenic assays: E_2_ (17β-estradiol) (Sigma Aldrich), luteolin (Extrasynthese, France), mangiferin (Sigma-Aldrich), isomangiferin (Chemos GmbH, Germany), protocatechuic acid, *p*-coumaric acid (Fluka Analytical, Sigma-Aldrich), scolymoside (Sigma-Aldrich), and iriflophenone-3-*C*-β-D-glucoside (Sigma-Aldrich). Phenolic compounds listed above were also used to identify and/or quantify compounds in the extract and fractions, in addition to phloretin-3',5'-di-*C*-β-D-glucoside (isolated from *C*. *subternata*, unpublished results), eriocitrin (Extrasynthese), hesperidin (Sigma-Aldrich), vicenin-2 (Phytolab, Germany), isorhoifolin (Extrasynthese), scolymoside (isolated from *C*. *subternata*, unpublished results), and iriflophenone-3-*C*-β-D-glucoside-4-*O*-β-D-glucoside (isolated from *Cyclopia genistoides* [49]).

### Fractionation of SM6Met

#### Preparation of SM6Met

The method (S1 Fig), executed at room temperature and adapted from Mfenyana *et al*. [31], entailed defatting of 500 g finely milled, dried plant material, originating from a *Cyclopia subternata* harvesting (M6; harvested on 30 March 2004 from a commercial plantation at Kanetberg farm near Barrydale, South Africa) [31], by stirred extraction over a period of 24 h, using 2 L of dichloromethane (Merck, South Africa, 99.0% pure). Following extraction the plant material was filtered, the filtrate discarded, and the plant material residue air-dried overnight in a fume cabinet at room temperature. This defatting process was repeated four times. Next, the air-dried, defatted M6 plant material was subjected to sequential extraction using three solvents (2 L each) in order of increasing polarity (ethyl acetate (99.5% pure), ethanol (99.5% pure) and methanol (99.0% pure), supplied by Merck). Each extraction step in the sequence was performed three times for 3 h per step. Before a solvent change was made, the plant material was air-dried overnight in a fume cabinet at room temperature. The filtrates of the methanol extraction step were retained, pooled, and the methanol evaporated under vacuum (Büchi Rotavap, Switzerland) at 40°C, where after a small quantity of deionized water was added and the extract freeze-dried (Virtis Advantage Plus, USA). The freeze dried extract was ground with a pestle and mortar until a fine powder was obtained, which was then stored under vacuum in a desiccator in the dark at room temperature. Four batches of SM6Met were prepared using the protocol above with the final SM6Met extract (311 g) being a homogenized mixture of the four batches.

#### Liquid-liquid fractionation

SM6Met (4.3 g), suspended in 300 mL deionized water and decanted into a separating funnel, was liquid-liquid fractionated (S1 Fig) using 150 mL of *n*-butanol (Sigma Aldrich, South Africa). Mixing was achieved by inverting the funnel 5 times (pressure in the separating funnel was released after every invert), where after the mixture was allowed to form two layers, and the lower, polar, and the upper, less polar, layers collected separately. For the purpose of this study the less polar fraction will be referred to as the non-polar fraction (NPF). The process was repeated four times. The resultant fractions, the polar fraction (PF) and the NPF, were pooled and rotary evaporated under vacuum at 40°C. Following freezing and freeze-drying, the dried extracts were stored at room temperature under vacuum in a desiccator in the dark.

#### High performance counter-current chromatography (HPCCC) fractionation

A multilayer coil planet J-type centrifuge Spectrum model (Dynamic Extraction, United Kingdom) equipped with two preparative (1.6 mm i.d.) and two analytical (0.8 mm i.d.) coils of polytetrafluoroethylene (PTFE) tubing, connected in series for a total volume of 172 ml, was used. The inner β_r_-value for the preparative coil was measured as 0.52 at the internal end of the coil and the outer β_r_-value was 0.86 (equation β_r_ = r/R, in this case r is defined as the distance from the coil (planetary) axis to the nearest and farthest layer of the PTFE tubes wound around the coil system), while the inner and outer β_r_-values for the analytical coil were 0.64 and 0.81, respectively. Fractionation was performed at 30°C and rotation speed of 1600 revolutions per min.

The two-phase solvent system used consisted of *tert*-butyl methyl ether (Sigma Aldrich)–*n*-butanol (Sigma Aldrich)–water (2:1:5, v/v). The solvents were mixed in a separating funnel, allowed to equilibrate at room temperature and separated into the organic and aqueous phases shortly before use. The separated phases were degassed using sonication. The upper organic phase served as the stationary phase, whereas the lower aqueous phase served as the mobile phase.

The column was filled with the stationary (organic) phase using a Gilson 305 HPLC pump (Gilson, USA). The sample (30 mg NPF) was dissolved in 2.5 mL organic phase and 2.5 mL aqueous phase and then injected into the coil using a manual sample injection valve and a 10 mL loop. The mobile (aqueous) phase was pumped in the head-to-tail direction at a flow rate of 3 mL/min. The effluent of the column, monitored between 210 nm and 400 nm, using a Waters 2996 diode-array detector (Waters, USA) equipped with a semi-preparative flow cell (3 mm path length), was collected into test tubes at 1 min intervals, using a Gilson FC203B fraction collector. The mobile phase flow was stopped at 52 min and the stationary phase pumped at 10 ml/min until 75 min. The procedure was repeated 15 times processing 450 mg NPF in total. The fractions collected were pooled into 3 main fractions, F1-F3, based on clustering of peaks observed on a chromatogram for the separation of the NPF constructed using maximum absorbance between 210 nm and 400 nm (S2 Fig). Fractions F1-F3 were rotary evaporated under vacuum at 40°C, frozen and freeze-dried, where after they were stored at room temperature under vacuum in a desiccator in the dark.

### Evaluation of estrogenic activity

#### Cell culture

HEK293 human embryonic kidney cells [50] (a kind gift from A. Swart, Stellenbosch University, ATCC cat# CRL-1573) and MCF-7BUS cells [51] (a kind gift from A. Soto, Tufts University, USA) were maintained in Dulbecco’s Modified Eagles’s Medium (DMEM) [Sigma Aldrich] supplemented with 100 IU/ml penicillin and 100 μg/ml streptomycin (Sigma Aldrich) and 10% fetal calf serum (FCS) or 5% heat inactivated FCS, [Highveld Biologicals, South Africa], respectively. All cells were maintained at 37°C, 5% CO_2_ and 95% relative humidity.

#### Promoter reporter studies

HEK293 cells were seeded at 4 x 10^6^ cells per plate in sterile cell-binding 10 cm plates (The Scientific Group, South Africa) with 1:1 DMEM:HamF12 (Life Technologies, South Africa) containing 10% FCS, 100 IU/ml penicillin and 100 μg/ml streptomycin, where after the cells were allowed to settle for 24 h. The cells were then rinsed with PBS (Thermo Scientific, South Africa), pre-heated to 37°C, to remove the phenol red and the medium changed to phenol red-free DMEM (Sigma-Aldrich) supplemented with 10% FCS, 100 IU/ml penicillin and 100 μg/ml streptomycin. The HEK293 cells were then transfected with a total amount of 6150 ng of plasmid DNA using the FugeneXtreme transfection reagent (Roche Applied Science, South Africa) according to the manufacturer’s instructions. Cells were transfected with an ERE containing promoter reporter construct (ERE.vit2.luc [52], a kind gift from K. Korach, National Institute of Environmental Health Science, USA) and either estrogen receptor (ER)α (pSG5-hERα [53],) or ERβ (pSG5-hERβ [52], gifts from F. Gannon, European Molecular Biology Laboratory, Heidelberg, Germany. For cells transfected with ERα, 150 ng pSG5-hERα, 3750 ng ERE.vit2.luc, and 2250 ng empty vector (pGL2-Basic, Promega, USA) were used, while for cells transfected with ERβ 150 ng pSG5-hERβ, 3000 ng ERE.vit2.luc, and 3000 ng empty vector were used.

After 24 h the cells were replated into sterile 24-well cell-binding tissue culture plates (Whitehead Scientific (Pty) Ltd, South Africa) at 5 x 10^4^ cells per well using phenol red-free DMEM supplemented with 10% charcoal stripped FCS (Highveld Biologicals), 100 IU/ml penicillin and 100 μg/ml streptomycin. The following day the cells were treated with the respective test compounds or samples (extracts or fractions), all dissolved in DMSO to yield a final DMSO concentration of 0.1% (v/v) in the medium. All extracts and fractions were tested at a concentration of 9.8 μg/mL [31].

The samples and test compounds were tested in both agonist and antagonist (in the presence of 10^−11^ M E_2_) mode, with DMSO serving as the solvent control, and E_2_, the endogenous ligand of the ER, serving as the positive control. After 24 h the cells were rinsed using 500 μL ice cold PBS and lysed overnight at -20°C using 50 μL passive lysis buffer [0.2% (vol/vol) Triton, 10% (vol/vol) glycerol, 2.8% (vol/vol) Tris-phosphate-EDTA, and 1.44 mM EDTA]. Luciferase assays were performed using the Luciferase assay reagent (Promega, USA) according to manufacturer’s instruction, where 50 μL of luciferase reagent was added to 10 μL of cell lysate. The luciferase activity, given in relative light units (RLUs), was obtained using a Veritas luminometer (Turner Biosystems, USA). Protein determination was done using the Bradford method [54], where 250 μL of the Bradford reagent was added to 5 μL of the cell lysate in a 96-well plate. Luciferase RLUs were normalised to protein concentration and the results were expressed as fold-induction relative to DMSO, which was set as one, for agonist mode, whereas, for antagonist mode the results were expressed as fold-induction relative to 10^−11^ M E_2_, which was set as one.

#### Proliferation studies

MCF-7 BUS cells, which had been withdrawn from the penicillin-streptomycin mixture for at least 7 days, were plated at a density of 1 x 10^4^ cells per well in a 96-well plate (Greiner Bio-One, Germany) in DMEM containing 5% FCS. The next day the cells were washed with PBS (200 μL/well), pre-heated to 37°C, and the medium was changed to unsupplemented phenol red-free DMEM (200 μL/well) for 24 h (steroid and growth factor starvation), after which the medium was aspirated and cells incubated for a further 24 h with the test compounds and extracts (in DMSO) in phenol red-free DMEM containing 10% stripped FCS. The medium was then aspirated and cells re-induced at 24 and 48 h after initial treatment. All assays included a negative control consisting of 0.1% (v/v) DMSO and a positive control (10^−9^ M E_2_). The MTT [3-(4,5-dimethylthiazolyl-2)-2,5-diphenyltetrazolium bromide] (Sigma Aldrich) assay was performed 24 h after the last treatment, where medium was aspirated and cells incubated with unsupplemented phenol red-free medium (200 μL/well) plus MTT solution (5 mg/mL; 50 μL/well) for 4 h at 37°C. After the 4 h incubation, the wells were aspirated and DMSO (solubilisation solution; 200 μL/well) was added. The DMSO was then pipetted up and down twice per well in order to obtain a uniform purple colour in each well. The absorbance value of each well was measured at 550 nm using a BioTek PowerWave 340 spectrophotometer (Biotek instruments, USA). Results were expressed as fold induction relative to the solvent control (DMSO) for agonism or relative to 10^-9^M E_2_ for antagonism, which was set as one. For investigation in agonist mode test samples were administered alone, whereas for investigation in antagonist mode, test samples were administered in the presence of 10^-9^M E_2_.

### Characterization of phenolic content

#### Quantification of phenolic compounds using qHPLC

Stock solutions of standards, SM6Met and fractions, were prepared in DMSO and frozen at -20°C until needed for analysis. For experimental analysis, the defrosted extract, fractions and standards were appropriately diluted with water and ascorbic acid (Sigma Aldrich) was added to a final concentration of ca 9 mg/mL. The mixtures were then filtered using Millex-HV syringe filters (Millipore, USA) with a 0.22 μm pore size. Separation was achieved on a Gemini-NX C18 column (150 × 4.6 mm; 3 μm; 110 Å; Phenomenex, USA), protected by a guard column (4 x 3.0 mm; Phenomenex) of the same stationary phase, with 2% aqueous acetic acid (A, Sigma Aldrich) and HPLC gradient-grade acetonitrile (B, Sigma Aldrich) as mobile phases. Injection volumes ranged from 10–20 μL for standards and 15 μL for the extract and fractions. Separation was performed using the HPLC method described by De Beer *et al*. [55]. A flow rate of 1 mL/min was used with the following mobile phase gradient: 0–2 min (8% B), 2–27 min (8–38% B), 27–28 min (38–50% B), 28–29 min (50% B), 29–30 min (50–8% B), 30–40 min (8% B).

The dihydrochalcones (phloretin-3',5'-di-*C*-β-D-glucoside and 3-hydroxyphloretin-3',5'-di-*C*-hexoside), flavanones (eriocitrin and hesperidin), benzophenones (iriflophenone-3-*C*-β-D-glucoside and iriflophenone-3-*C*-β-D-glucoside-4-*O*-β-D-glucoside) and benzoic acid (protocatechuic acid) were quantified at 288 nm, whereas the xanthones (mangiferin and isomangiferin), flavones (luteolin and scolymoside) and hydroxycinnamic acid (*p*-coumaric acid) were quantified at 320 nm. A 7-point calibration curve was set up for all the available standards: luteolin, mangiferin, isomangiferin, eriocitrin, hesperidin, vicenin-2, iriflophenone-3-*C*-β-D-glucoside-4-*O*-β-D-glucoside, iriflophenone-3-*C*-β-D-glucoside, scolymoside, phloretin-3',5'-di-*C*-β-D-glucoside, protocatechuic acid and *p*-coumaric acid. 3-Hydroxyphloretin-3',5'-di-*C*-hexoside were expressed in terms of phloretin-3',5'-di-*C*-β-D-glucoside equivalents as no authentic reference standard was available.

#### Identification of phenolic compounds using LC-MS/MS analysis

Analyses were performed on a Waters Acquity ultra-performance liquid chromatography (UPLC) system comprising an in-line degasser, diode array detector (DAD), column oven and binary pump. This system was coupled to a Synapt G2 quadrupole time of flight (QTOF) mass spectrometer (Waters) containing an electrospray ionization (ESI) source. The method used was the same as described in De Beer *et al*. [55] for LC-MS/MS analysis. An injection volume of 10 μL was used for samples with the eluent being split 1:1 before introduction to the ionization source. MS results were acquired in the negative ionization mode with MS and MS^E^ for each sample. For MS^E^ a collision energy ramp from 25 to 60 V was used, whereas for MS/MS data a collision energy of 30 V was used. The parameters used for MS were as follows: capillary voltage, 2.5 kV, cone voltage 15 V, desolvation temperature 275°C, source temperature 120°C and nitrogen flow rate 650 L/h. Peaks were identified by comparing LC-MS spectra, UV-Vis spectra and retention times to authentic reference standards or as in De Beer *et al*. [55]. Data were acquired and processed by means of MassLynx v4.1 software (Waters).

### Data manipulation and statistical analysis

GraphPad Prism version 5 (GraphPad Software, USA) was used for graphical representation and statistical analysis of experimental data. One-way analysis of variance (ANOVA) was performed with Dunnett’s multiple comparisons test as post-test. For all experiments, unless otherwise indicated, the error bars represent the SEM (standard error of means) of three independent experiments done in triplicate.

## Results

The desirable estrogenic attributes previously identified [31,32] were used as the basis for the activity-guided fractionation of SM6Met. Specifically, ERα antagonism and ERβ agonism activity were evaluated in the HEK293 cell line (does not contain endogenous ERα or ERβ (S3A Fig) transfected with an ERE-containing promoter reporter construct and expression vectors for either ERα or ERβ. In addition, antagonism of E_2_-induced breast cancer cell proliferation was investigated in MCF-7 BUS cells, which endogenously express both ERα and ERβ (Fig B in S3 Fig), and is a more complex and physiologically relevant model. Furthermore, LC-MS and qHPLC were used to track major polyphenols during fractionation to allow for correlation with estrogenic activity in an attempt to identify possible marker compounds.

**Fig 1:**
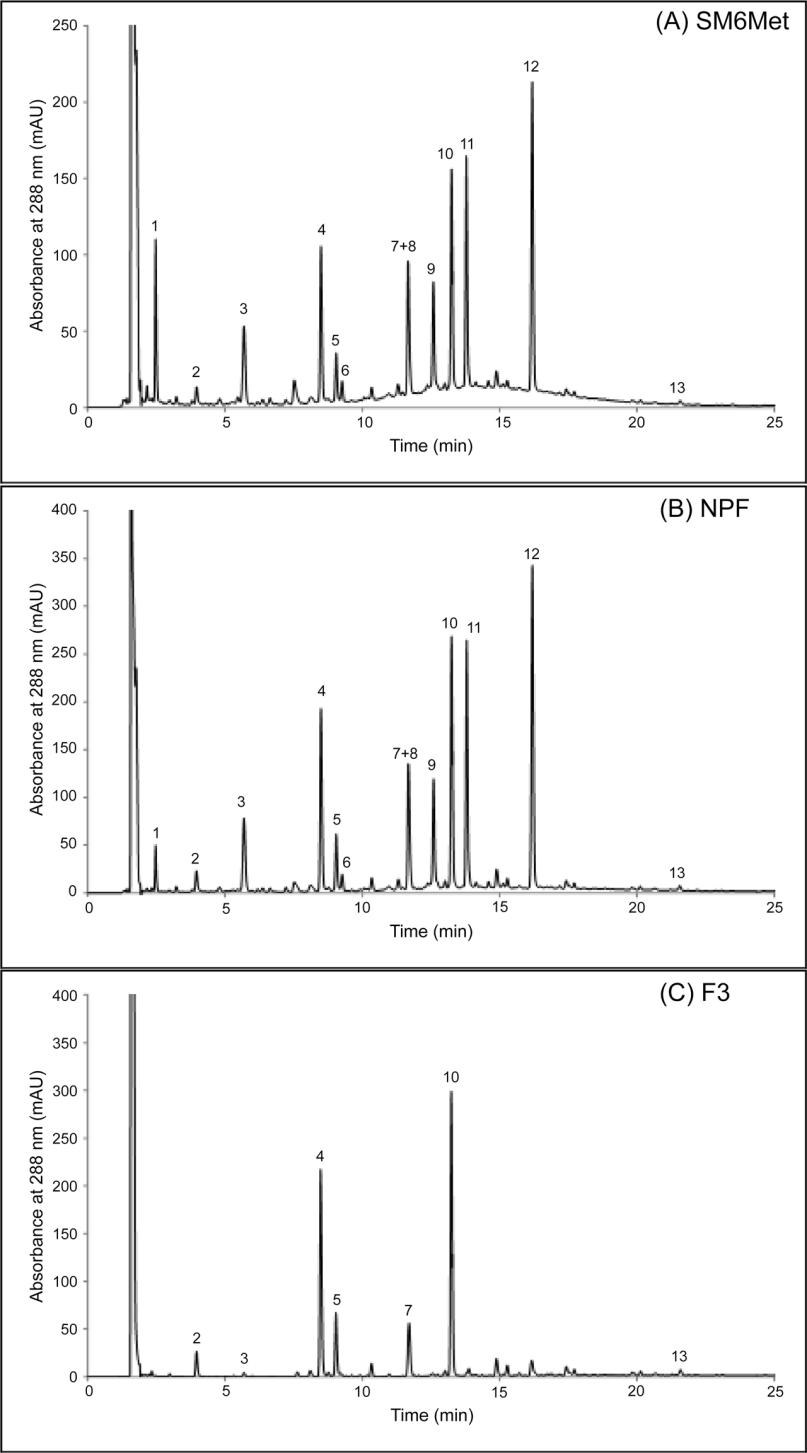
Identification of the major phenolic constituents in SM6Met, the NPF, and F3. qHPLC chromatograms of (A) SM6Met, (B) the NPF, and (C) F3. The first peak in all samples represents ascorbic acid (not numbered), which was added to prevent oxidation. Peak 1 = iriflophenone-3-*C*-β-D-glucoside-4-*O*-β-D-glucoside; 2 = protocatechuic acid; 3 = iriflophenone-3-*C*-β-D-glucoside; 4 = mangiferin; 5 = isomangiferin; 6 = vicenin-2; 7 = *p*-coumaric acid; 8 = 3-hydroxyphloretin-3',5'-di-*C*-hexoside;9 = eriocitrin; 10 = scolymoside; 11 = phloretin-3',5'-di-*C*-β-D-glucoside; 12 = hesperidin; 13 = luteolin.

To assist the reader we provide an overview of the fractionation process and yields obtained (S1 Fig). Initially, the M6 harvesting of *C*. *subternata* was used to produce SM6Met, which was subsequently fractionated into the PF and the NPF using liquid-liquid fractionation. The NPF was sub-fractionated via HPCCC yielding F1-F3. The qHPLC chromatograms of SM6Met (Fig 1A), the NPF (Fig 1B) and F3 (Fig 1C), with their respective major polyphenolic constituents, are shown in Fig 1.

### Liquid-liquid fractionation of SM6Met to obtain a polar fraction (PF) and a non-polar fraction (NPF)

SM6Met (4.30 g), displaying the three desirable estrogenic traits of interest [31,32], was fractionated, according to relative polarity by means of liquid-liquid fractionation (S1 Fig), into two fractions, the PF (2.92 g) and the NPF (1.21 g), resulting in a total recovery of 96%.

#### Constituent analyses indicate that the major compounds in SM6Met are retained and concentrated in the NPF

LC-MS/MS analysis was used as a sensitive technique to identify compounds present in SM6Met (S1 Table, Fig A in S4 Fig). Most of the identified compounds had previously been identified in aqueous *C*. *subternata* extracts [55] or isolated from *C*. *subternata* methanol extracts [56]. However, some new compounds present at low concentrations were tentatively identified namely, luteolin-*O*-hexoside, chrysoeriol-*O*-deoxyhexose-*O*-hexoside (flavone), and quercetin-*O*-deoxyhexose-*O*-hexoside (flavonol).

The major phenolic compounds in SM6Met identified by LC-MS/MS (S1 Table), i.e. iriflophenone-3-*C*-β-D-glucoside, mangiferin, isomangiferin, scolymoside, hesperidin, eriocitrin, phloretin-3',5'-di-*C*-β-D-glucoside, and 3-hydroxyphloretin-3',5'-di-*C*-hexoside, as well as the phenolic acid, protocatechuic acid, were quantified by qHPLC (Fig 1A, Table 1). *p*-Coumaric acid was identified, but could not be accurately quantified due to co-elution. In addition, luteolin, a minor compound, was also quantified as previous studies had shown that the presence of luteolin might correlate with estrogenic activity of *Cyclopia* extracts [30,31]. Furthermore, two other minor compounds in SM6Met, iriflophenone-3-*C*-β-D-glucoside-4-*O*-β-D-glucoside and vicenin-2, were included as they could be quantified.

**Table 1:** Quantification of major and some minor phenolic compounds in SM6Met, PF and NPF as determined by qHPLC.

		SM6Met			PF			NPF		
#	Polyphenol	g/100g^a^	Concentration factor^b^	Yield (%)^c^	g/100g	Concentration factor	Yield (%)	g/100g	Concentration factor	Yield(%)
**4** ^**d**^	**Mangiferin**	1.90	1.00	100	0.09	0.05	3.22	5.56	2.93	82.35
**5**	**Isomangiferin**	0.65	1.00	100	0.05	0.08	5.22	1.88	2.91	81.39
**6**	**Vicenin-2 (apigenin-6,8-di-C-β-D-glucoside)**	0.19	1.00	100	0.10	0.53	35.74	0.37	1.95	54.80
**10**	**Scolymoside (luteolin-7-*O*-rutinoside)**	2.87	1.00	100	0.24	0.08	5.68	8.25	2.88	80.89
**13**	**Luteolin**	0.04	1.00	100	Nd^e^	Nd	0.00	0.11	2.63	77.39
**1**	**Iriflophenone-3-*C*-β-D-glucoside-4-*O*-β-D-glucoside**	0.88	1.00	100	1.02	1.16	78.70	0.60	0.68	19.19
**3**	**Iriflophenone-3-*C*-β-D-glucoside**	0.58	1.00	100	0.20	0.34	23.41	1.36	2.34	65.98
**8**	**3-Hydroxy-phloretin-3',5'-di-*C*-hexoside**	1.16	1.00	100	0.38	0.32	22.24	2.79	2.41	67.68
**11**	**Phloretin-3',5'-di-*C-*β-D-glucoside**	1.77	1.00	100	0.35	0.20	13.43	4.79	2.70	76.15
**9**	**Eriocitrin (eriodictyol-7-*O*-rutinoside)**	0.85	1.00	100	0.19	0.22	15.18	2.13	2.52	70.52
**12**	**Hesperidin (hesperetin-7-*O*-rutinoside)**	2.05	1.00	100	0.37	0.18	12.26	5.36	2.61	73.58
**7**	***p*-Coumaric acid**	co-el^f^	1.00	co-el	Nd	Nd	0.00	co-el	co-el	co-el
**2**	**Protocatechuic acid**	0.11	1.00	100	Nd	Nd	0.00	0.35	3.07	89.54

^a^g/100 g refers to the amount of the compound present (g) in 100 g extract/fraction.

^b^Concentration factor refers to the amount of a compound in an extract or fraction divided by the amount of the compound in SM6Met. For example, for mangiferin in the PF the concentration factor is (0.09 g/1.90 g) = 0.05.

^c^Yield was calculated relative to the initial concentration of a compound in SM6Met, which was set to 100%. For example, for mangiferin in the PF, the yield is (0.061 g/1.90 g) x 100 = 3.22%, where 0.061 g (0.09 g x 67.9 g/100 g) refers to the total amount of mangiferin present in the PF as only 67.9 g of the PF is obtained during fractionation of 100 g SM6Met (as illustrated in S1 Fig).

^d^Refers to peak number in qHPLC chromatogram in Fig 1.

^e^Nd—polyphenols were not detected due to absence or trace amounts.

^f^co-el—Co-elution refers to the fact that *p*-coumaric acid co-elutes with 3-hydroxyphloretin-3',5'-di-*C*-hexoside.

The flavanones, hesperidin and eriocitrin, the flavones, scolymoside and luteolin, and the dihydrochalcones, 3-hydroxyphloretin-3',5'-di-*C*-hexoside and phloretin-3',5'-di-*C*-β-D-glucoside, comprised approximately 24% each of the quantified major compounds. The xanthones, mangiferin and isomangiferin, constituted 21% of the quantified major compounds. Furthermore, the benzophenone, iriflophenone-3-*C*-β-D-glucoside, comprised only 4.8% of the quantified major compounds, while the phenolic acids, *p*-coumaric acid and protocatechuic acid, were only present in trace amounts.

LC-MS/MS results (S1 Table, S4B and S4C Fig) showed that all of the major compounds in SM6Met were retained as major compounds in the NPF, whereas for the PF most of these major compounds, with the exception of 3-hydroxyphloretin-3',5'-di-*C*-hexoside, scolymoside, phloretin-3',5'-di-*C*-β-D-glucoside, and hesperidin, were now present as minor compounds. In addition, qHPLC analysis (Table 1) of the NPF, when compared to SM6Met, showed an overall 2- to 3-fold increase in the concentration factor of the major compounds, while a 3- to 20-fold decrease was observed in the PF. In terms of the overall yield of the quantified compounds, relative to SM6Met (set as 100%), values ranged from 66–90% for the NPF and 0.0–23% for the PF (Table 1).

Further analysis of the NPF (Table 1) showed that the xanthones, flavones, flavanones and dihydrochalcones each contributed 23–26% towards the quantified compounds, whereas the benzophenone, iriflophenone-3-*C*-β-D-glucoside, and the phenolic acid, protocatechuic acid, contributed 4.2 and 1.1%, respectively. Thus, as a percentage of the quantified compounds the xanthones, flavones, and protocatechuic acid slightly increased, while iriflophenone-3-*C*-β-D-glucoside, flavanones, and dihydrochalcones slightly decreased in the NPF relative to SM6Met. In contrast, although in much lower quantities, the PF consisted mostly of the dihydrochalcones, 3-hydroxyphloretin-3',5'-di-*C*-hexoside, and phloretin-3',5'-di-*C*-β-D-glucoside (39.0%), and the flavanones (30.0%), followed by the flavone, scolymoside (12.8%), the benzophenone, iriflophenone-3-*C*-β-D-glucoside (10.7%), and low amounts of the xanthones (7.5%).

In conclusion, constituent analysis using qHPLC indicates that liquid-liquid fractionation of SM6Met results in retention and concentration (2- to 3-fold) of the major compounds in the NPF.

#### Estrogenic activity analyses indicate that the desirable estrogenic attributes of SM6Met are retained in the NPF

**Fig 2:**
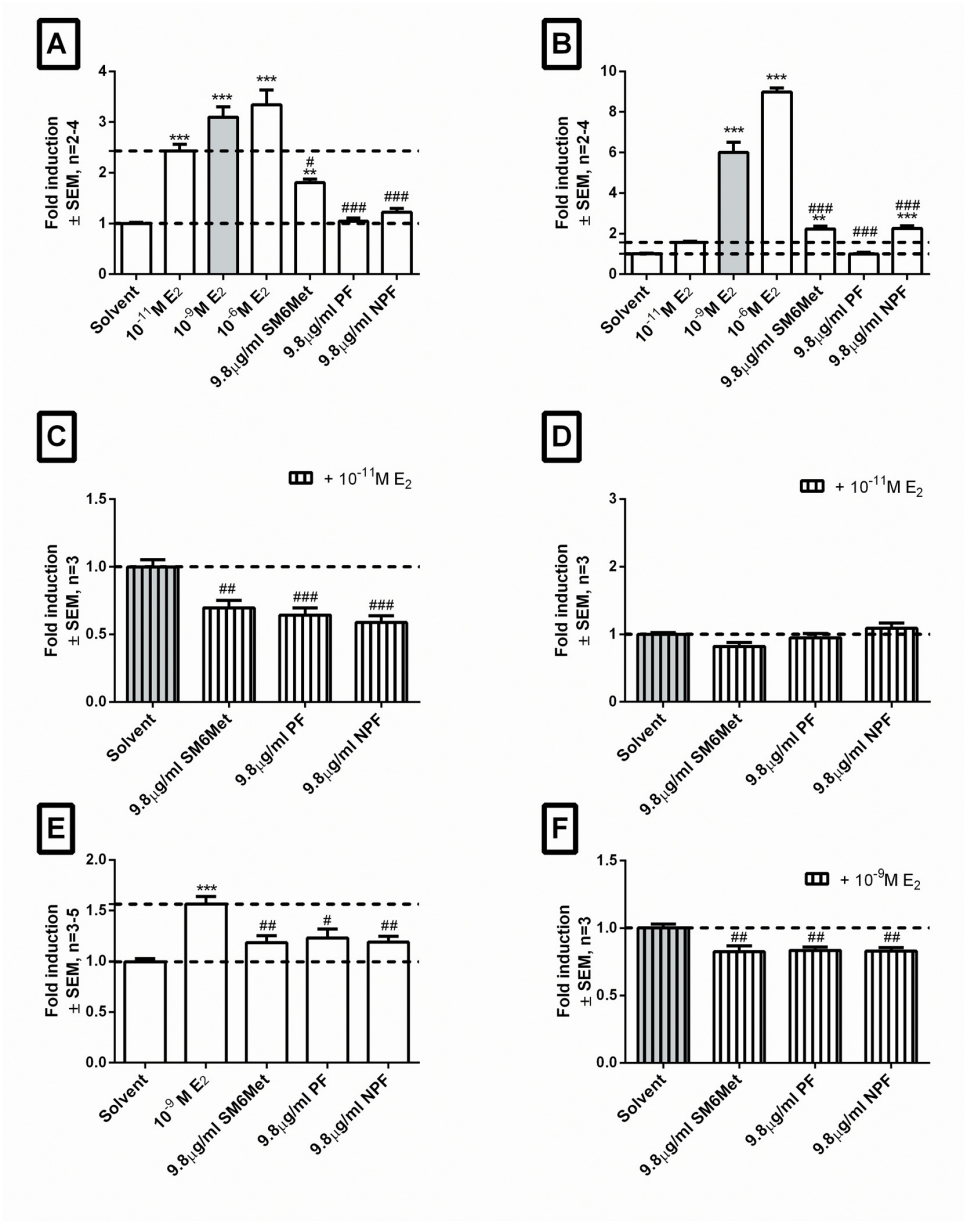
The NPF retains all three desirable estrogenic attributes of SM6Met. To establish retention of the desirable estrogenic attributes of SM6Met, promoter reporter and proliferation studies were conducted using E_2_ (positive control), SM6Met, the NPF, and the PF. Promoter reporter studies in HEK293 cells transfected with ERE.vit2.luc and either pSG5-hERα (A&C) or pSG5-hERβ (B&D) were conducted in agonist (A&B) and antagonist (C&D) mode. Proliferation studies using MCF-7 BUS cells were conducted in agonist (E) and antagonist (F) mode. Statistical analysis was done using One-way ANOVA with Dunnett’s post-test comparing all columns to either solvent control (black bar) (*, P<0.05; **, P<0.01;***, P<0.001) or E_2_ (grey bar) (#, P<0.05; ##, P<0.01; ###, P<0.001). The dotted lines through the bars represent the values for solvent control and/or E_2_ used for statistical analysis.

One of the three desirable estrogenic attributes of SM6Met previously demonstrated in COS-1 cells [32], namely ERα antagonism, was also demonstrated in HEK293 cells (Fig 2C), however, in contrast to previous results, significant agonism via ERα was also shown in HEK293 cells (Fig 2A). This conflict with previous results may be explained by the fact that the HEK293 cells displayed a greater estrogenic sensitivity than the COS-1 cells [32], as demonstrated by the differences in response to E_2_. However, SM6Met-induced ERα agonism was significantly lower than that of 10^-11^M E_2_ (Fig 2A). Both the PF and the NPF retained significant ERα antagonist activity (Fig 2C), one of the positive estrogenic attributes of interest. In addition, in contrast to SM6Met, which displayed significant ERα agonism (Fig 2A), neither the PF nor the NPF showed significant agonism via ERα (Fig 2A). The absence of ERα agonism, as seen for the PF and the NPF, positions them more favorably in terms of positive estrogenic attributes compared to the original SM6Met as ERα agonism is known to be pro-proliferative [42].

The second desired positive estrogenic attribute we investigated was ERβ agonism. SM6Met showed significant ERβ agonism (Fig 2B), which was also significantly higher than that of 10^-11^M E_2_. These results suggest that SM6Met is a stronger ERβ agonist than an ERα agonist. Furthermore, SM6Met did not display ERβ antagonism (Fig 2D). ERβ agonism was retained by the NPF, however, the PF displayed no significant ERβ agonism. In addition, no ERβ antagonism was seen for either fraction (Fig 2D).

The third and final positive estrogenic attribute investigated was antagonism of E_2_-induced breast cancer cell proliferation in MCF-7 BUS cells. As previously demonstrated [32], SM6Met showed significant antagonism (Fig 2F) of E_2_-induced breast cancer cell proliferation. In addition, it did not induce breast cancer cell proliferation in agonist mode (Fig 2E). Furthermore, significant antagonism of E_2_-induced breast cancer cell proliferation was retained by both the PF and the NPF (Fig 2F). In addition, like SM6Met, PF and NPF did not induce significant breast cancer cell proliferation in agonist mode (Fig 2E).

Thus in summary, the NPF retained all of the positive estrogenic attributes previously described for SM6Met, namely, ERα antagonism, ERβ agonism and antagonism of E_2_-induced breast cancer cell proliferation, whereas PF retained only ERα antagonism and antagonism of E_2_-induced breast cancer cell proliferation, but not ERβ agonism. As ERβ agonism is considered an important positive estrogenic attribute for the development of a possible phytoestrogenic nutraceutical, due to its anti-proliferative function [42], we decided to focus on NPF, rather than PF, for subsequent fractionation.

### Fractionation of the NPF by HPCCC to obtain F1-F3

As the NPF retained all of the positive estrogenic attributes of interest, it was fractionated further using HPCCC, which yielded three major fractions (F1, F2 and F3), with 0.45 g of the NPF resulting in 0.180 g F1, 0.043 g F2 and 0.147 g F3 (S1 and S2 Figs). The overall yield of these three HPCCC fractions was 82.2%.

#### Constituent analysis indicates that HPCCC fractionation of the NPF results in the divergence of the major phenolic compounds across F1, F2, and F3

LC-MS/MS analysis of the three HPCCC fractions (S1 Table, Fig D–F in S4 Fig) revealed that each HPCCC fraction had a different compound profile, which was also different from that of the NPF. In addition, qHPLC analysis of the HPCCC fractions (Table 2, Fig 1C) revealed that F1 consisted mainly of dihydrochalcones (90% of quantified compounds in F1) and retained most (97%) of the dihydrochalcones recovered during HPCCC (Table 2). Of the dihydrochalcones in F1, phloretin-3',5'-di-*C*-β-D-glucoside was concentrated approximately 5-fold and 2-fold relative to SM6Met and the NPF, respectively, while 3-hydroxyphloretin-3',5'-di-*C*-hexoside was concentrated approximately 2-fold relative to SM6Met, but not concentrated relative to the NPF (Tables 1 and 2). F1 also contained a minor flavanone component (10% of quantified compounds in F1) consisting mostly of eriocitrin.

**Table 2:** Quantification of major and some minor phenolic compounds in F1, F2 and F3 as determined by qHPLC.

		F1			F2			F3		
#	Polyphenol	g/100g^a^	Concentration factor^b^	Yield (%)^c^	g/100g	Concentration factor	Yield (%)	g/100g	Concentration factor	Yield (%)
**4** ^**d**^	**Mangiferin**	Nd^e^	Nd	0.00	Nd	Nd	0.00	11.57	6.09	55.96
**5**	**Isomangiferin**	Nd	Nd	0.00	Nd	Nd	0.00	4.02	6.18	56.84
**6**	**Vicenin-2 (apigenin-6,8-di-C-β-D-glucoside)**	0.65	3.42	38.52	Nd	Nd	0.00	Nd	Nd	0.00
**10**	**Scolymoside (luteolin-7-*O*-rutinoside)**	Nd	Nd	0.00	Nd	Nd	0.00	17.05	5.94	54.60
**13**	**Luteolin**	Nd	Nd	0.00	Nd	Nd	0.00	0.21	5.25	48.25
**1**	**Iriflophenone-3-*C*-β-D-glucoside-4-*O*-β-D-glucoside**	1.16	1.32	14.84	Nd	Nd	0.00	Nd	Nd	0.00
**3**	**Iriflophenone-3-*C*-β-D-glucoside**	Nd	Nd	0.00	10.28	17.72	47.68	0.08	0.14	1.27
**8**	**3-Hydroxy-phloretin-3',5'-di-*C*-hexoside**	2.78	2.40	26.99	0.40	0.34	0.93	Nd	Nd	0.00
**11**	**Phloretin-3',5'-di-*C-*β-D-glucoside**	8.42	4.76	53.56	0.96	0.54	1.46	Nd	Nd	0.00
**9**	**Eriocitrin (eriodictyol-7-*O*-rutinoside)**	1.19	1.40	15.76	11.15	13.12	35.29	Nd	Nd	0.00
**12**	**Hesperidin (hesperetin-7-*O*-rutinoside)**	0.08	0.04	0.44	39.45	19.24	51.77	Nd	Nd	0.00
**7**	***p*-Coumaric acid**	Nd	Nd	0.00	Nd	Nd	0.0	0.57	co-el^f^	co-el
**2**	**Protocatechuic acid**	Nd	Nd	0.00	Nd	Nd	0.0	0.65	5.91	54.30

^a^g/100 g refers to the amount of the compound present (g) in 100 g extract/fraction.

^b^Concentration factor refers to the amount of a compound in a extract or fraction divided by the content of the compound in SM6Met for example mangiferin in F3 (11.57 g/1.90 g) = 6.09.

^c^Yield was calculated relative to the initial concentration of a compound in SM6Met, which was set to 100%. For example, for mangiferin in F3, the yield is (1.063 g/1.90 g) x 100 g = 55.96%, where 1.063 g (11.57 g x 9.19 g/100 g) refers to the total amount of mangiferin present in F3 as only 9.19 g of F3 is obtained during fractionation of 100 g SM6Met (as illustrated in S1 Fig).

^d^Refers to peak number in qHPLC chromatogram in Fig 1.

^e^Nd—polyphenols were not detected due to absence or trace amounts

^f^Co-elution refers to the fact that *p*-coumaric acid co-elutes with 3-hydroxyphloretin-3',5'-di-*C*-hexoside where present.

In contrast to F1, F2 retained 91% of all flavanones recovered during HPCCC (Table 2) and consisted mostly of the flavanones (81% of quantified compounds in F2). Of the flavanones in F2, eriocitrin was concentrated approximately 13-fold and 5-fold relative to SM6Met and the NPF, respectively, while hesperidin was concentrated approximately 19-fold and 7-fold relative to SM6Met and the NPF, respectively (Tables 1 and 2). Although the benzophenone, iriflophenone-3-*C*-β-D-glucoside, only constituted approximately 16% of the quantified compounds in F2 it represented 97% of the benzophenones recovered during HPCCC and was concentrated approximately 18-fold and 8-fold relative to SM6Met and the NPF, respectively (Tables 1 and 2). F2 also contained a minor dihydrochalcone component (2% of quantified compounds in F2).

All the xanthones, flavones and phenolic acids eluted in F3 so that this fraction consisted of approximately 46% xanthones, 51% flavones and 4% phenolic acids (Table 2). Only trace amounts of the benzophenone, iriflophenone-3-*C*-β-D-glucoside, were present in F3. The xanthone component of F3 was concentrated approximately 6-fold and 2-fold relative to SM6Met and the NPF, respectively, while the flavones were concentrated approximately 5- to 6-fold and 2- to 3-fold relative to SM6Met and the NPF, respectively (Tables 1 and 2).

In terms of the overall yields of the quantified compounds, with respect to SM6Met (set as 100%), the dihydrochalcones in F1 values ranged from 27 to 54%, the flavanones in F2 values ranged from 35% to 52%, while the xanthones and flavones in F3 values ranged from 48% to 57% (Table 2).

To conclude, constituent analysis using qHPLC indicates that HPCCC fractionation of the NPF resulted in a dihydrochalcone-rich fraction (F1), a benzophenone- and flavanone-rich fraction (F2), and a xanthone- and flavone-rich fraction (F3). Thus, HPCCC fractionation resulted in the divergence of the type of phenolic compounds present in the NPF.

#### Estrogenic activity analysis reaffirms the divergence observed during constituent analysis in that the desirable estrogenic attributes of the NPF were also separated

**Fig 3:**
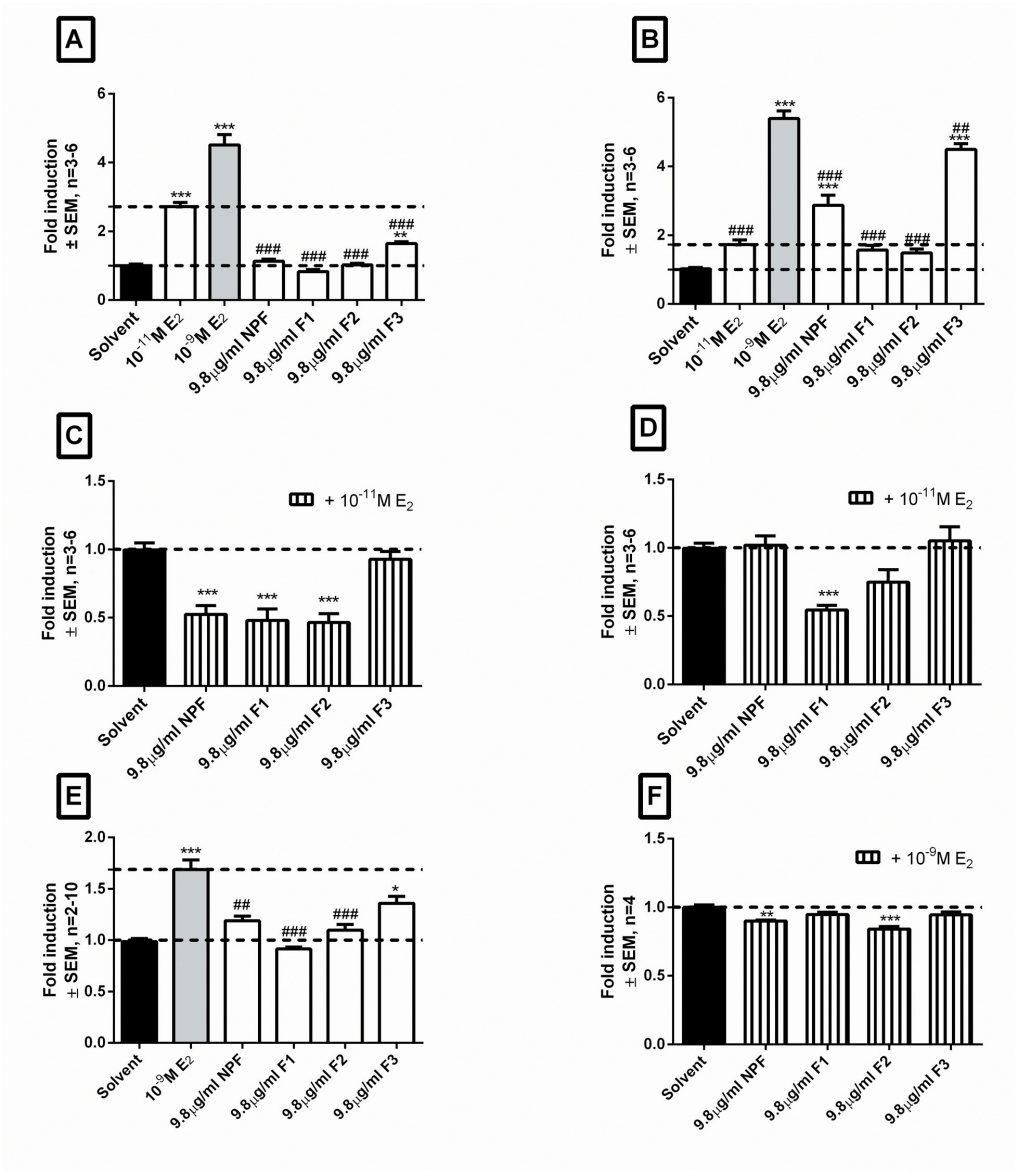
The three desirable estrogenic attributes of the NPF are not retained in one fraction after CCC fractionation. To establish retention of the desirable estrogenic attributes of the NPF, promoter reporter and proliferation studies were conducted using E_2_ (positive control), the NPF, F1, F2, and F3. Promoter reporter studies in HEK293 cells transfected with ERE.vit2.luc and either pSG5-hERα (A&C) or pSG5-hERβ (B&D) were conducted in agonist (A&B) and antagonist (C&D) mode. Proliferation studies using MCF-7 BUS cells were conducted in agonist (E) and antagonist (F) mode. Statistical analysis was done using One-way ANOVA with Dunnett’s post-test comparing all columns to either solvent control (black bar) (*, P<0.05; **, P<0.01;***, P<0.001) or E_2_ (grey bar) (#, P<0.05; ##, P<0.01; ###, P<0.001). The dotted lines through the bars represent the values for solvent control and/or E_2_ used for statistical analysis.

In accordance with the activity of the NPF, F1 and F2 displayed significant ERα antagonism, however, F3 displayed no ERα antagonism (Fig 3C). In addition, only F3 displayed significant ERα agonism (Fig 3A), though, it was weak and significantly lower than that of 10^−11^ M E_2_, reflecting an “activity profile” quite similar to that of SM6Met (Fig 2A).

Investigation of HPCCC fractions for ERβ agonism revealed that F3 acted as a very potent ERβ agonist, eliciting a 4.5-fold induction (Fig 3B). In contrast, F1 and F2 showed no significant ERβ activation. In addition, only F1 displayed significant ERβ antagonism (Fig 3D).

Similar to the NPF, none of the HPCCC fractions, with the exception of F3, induced significant breast cancer cell proliferation (Fig 3E). Furthermore, only F2, like the NPF, displayed significant antagonism of E_2_-induced breast cancer cell proliferation (Fig 3F).

Thus, in summary, analysis of the HPCCC fractions revealed divergence of the previously demonstrated positive estrogenic attributes of the NPF into separate fractions, with F1 and F2 retaining ERα antagonism, only F2 retaining antagonism of E_2_ induced breast cancer cell proliferation, and only F3 retaining ERβ agonist activity. ERβ agonism was the only desirable estrogenic attribute of the NPF augmented by HPCCC fractionation in that F3 induction via ERβ was significantly higher than that obtained with the NPF (statistics not shown). In addition, the ERβ agonism displayed by F3 was very robust in being significantly higher than that of 10^−11^ M E_2_ and comparable to that of 10^−9^ M E_2_ (4.5-fold vs. 5.4-fold). Furthermore, as ERβ agonists have recently come to the fore as strong alternative candidates for the treatment of breast cancer [40,57,58], F3 was chosen to be re-engineered [48].

### Reengineering of F3 with pure compounds (F3R) did not replicate ERβ agonist activity

It has been suggested that one of the best strategies for verifying the pharmacological contribution of individual compounds towards the activity of a mixture would be to reengineer the mixture using the compounds identified in the mixture [48]. Therefore, as quantitative data regarding several compounds in F3 was available (Table 2), the F3 fraction could be re-engineered as F3R using authentic reference standards of purity > 95%. F3R contained all the major identifiable compounds (S1 Table, Fig F in S4 Fig) plus two commercially available minor compounds that could be quantified (Table 2 and S1 Table, Fig F in S4 Fig) at the individual concentrations of these compounds in a 9.8 μg/ml solution of F3.

**Fig 4:**
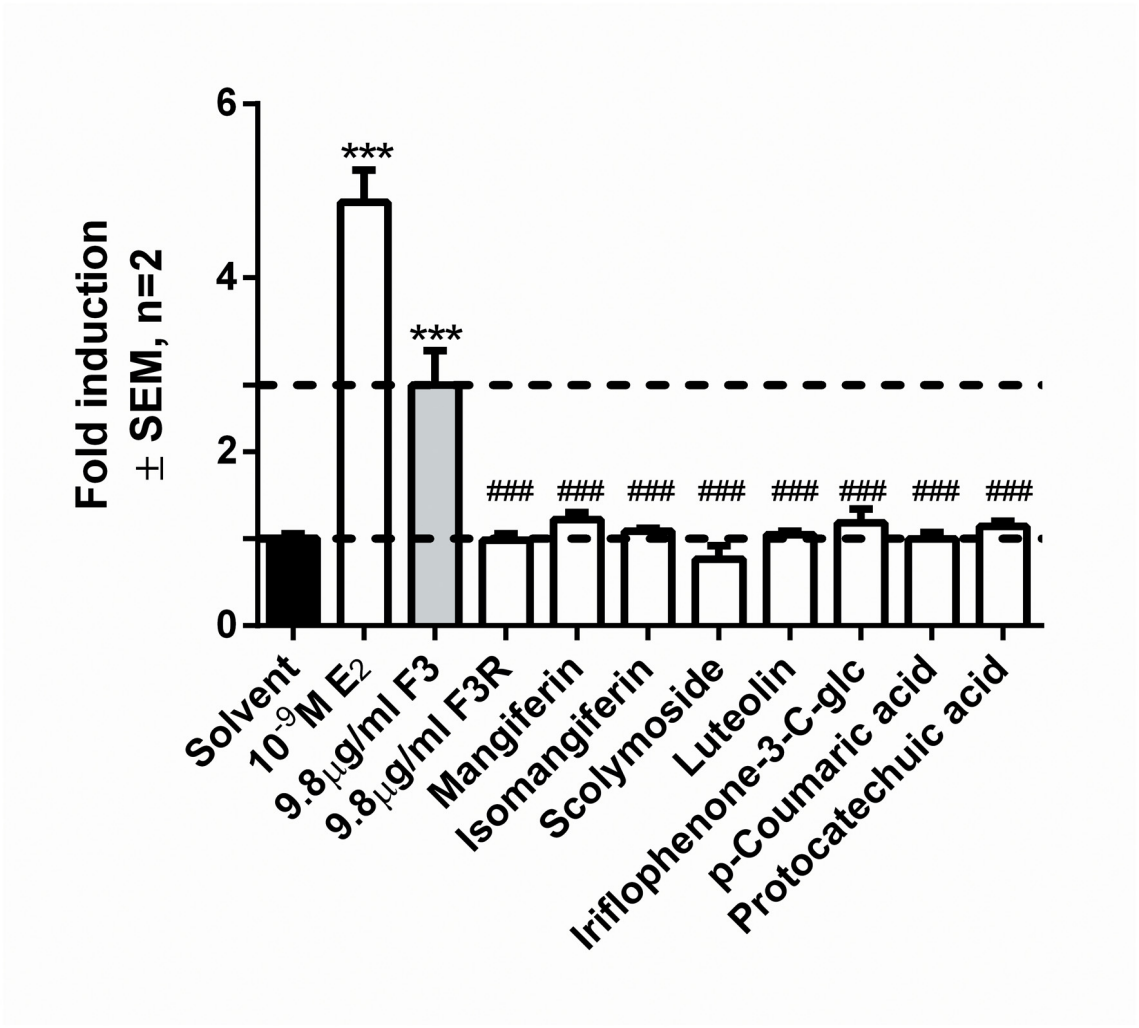
Reengineering of F3 with pure compounds (F3R) does not replicate ERβ agonist activity. To replicate the desirable estrogenic attribute displayed by F3, ERβ agonism, promoter reporter studies were conducted in HEK293 cells transfected with ERE.vit2.luc and pSG5-hERβ. Cells were induced with 10^−9^ M E_2_, 9.8 μg/mL F3, 9.8μg/mL F3R (consisting of 1.13 μg mangiferin, 0.39 μg isomangiferin, 1.67 μg scolymoside, 0.02 μg luteolin, 0.01 μg iriflophenone-3-*C*-glucoside, 0.06 μg *p*-coumaric acid, 0.06 μg protocatechuic acid as in 9.8 μg F3 (Table 2). Statistical analysis was done using One-way ANOVA with Dunnett’s post-test comparing all columns to either solvent control (black bar) (*, P<0.05; **, P<0.01;***, P<0.001) or F3 (grey bar) (#, P<0.05; ##, P<0.01; ###, P<0.001). The dotted lines through the bars represent the values for solvent control or F3.

F3 previously showed potent ERβ agonist activity (Fig 3B), which was also displayed upon re-investigation (Fig 4), however, F3R displayed no ERβ agonist activity. Furthermore, evaluation of the ERβ agonist activity of each individual compound at the concentration present in 9.8 μg/ml F3 also displayed no ERβ agonist activity.

Together these results indicate that the desired ERβ agonist activity of F3 could not be replicated by reengineering F3 with the available compounds (Table 2). This suggests that other compounds present in F3 may contribute to the robust ERβ activity of F3 (Fig F in S4 Fig).

## Discussion

Activity-guided fractionation is commonly used to identify compounds within mixtures responsible for specific activity profiles [57,59,60]. In the field of estrogenic nutraceuticals, activity-guided fractionation was most successfully employed to isolate the ERβ-subtype specific agonist, liquiritigenin, from MF101, a plant extract containing 22 herbs, which has ERβ selective properties [57]. In the case of SM6Met the desired positive estrogenic attributes are more complex in that ERβ agonism, ERα antagonism and antagonism of E_2_-induced breast cancer cell proliferation were simultaneously targeted. The existence of a compound displaying more than one desired estrogenic attribute seemed possible as the R,R enantiomer of 5,11-*cis*-diethyl-5,6,11,12-tetrahydrochrysene-2,8-diol (R,R-THC) has been shown to be both an ERα agonist and an ERβ antagonist [61–63].

Our results from liquid-liquid fractionation of SM6Met into the PF and the NPF seemed promising as indeed the three desirable estrogenic attributes were retained in the NPF (S2 Table). However, evaluation of the HPCCC fractions (F1, F2, F3) obtained from the NPF suggested that it is unlikely that the three desirable estrogenic attributes of SM6Met can be ascribed to a single compound. Specifically, it was shown that ERβ agonism was only observed for F3, ERα antagonism was only observed for F1 and F2, while antagonism of E_2_-induced breast cancer cell proliferation was only observed for F2 (S2 Table). Of note, ERβ agonism was significantly augmented during the fractionation process so that F3 displayed an approximately 2-fold increase in ERβ agonism relative to the starting material, SM6Met (S2 Table). Remarkably, the ERβ agonism of F3 was 260% more than that obtained with 10^−11^ M E_2_ and only 17% less than that obtained with 10^−9^ M E_2_.

The divergence of the three desirable estrogenic attributes observed during HPCCC fractionation may be ascribed to the separation of the major compounds into the individual HPCCC fractions. Specifically, F1 consisted mainly of the dihydrochalcones, F2 mainly of the flavanones and the benzophenone, and F3 mainly of the xanthones, the flavones, and the phenolic acids. In terms of identifying polyphenol marker compounds contributing to the estrogenic activity of F3, the fraction displaying robust ERβ agonism, it is unlikely that mangiferin or *p*-coumaric acid directly contributed as they lack estrogenic activity [29,30,64,65]. The other major xanthone present in F3, isomangiferin, has, to our knowledge, not been tested for estrogenic activity, nor has the flavone, scolymoside, or the phenolic acid, protocatechuic acid [33]. Thus, the flavone, luteolin, is the only compound in F3 that could have contributed to the estrogenic activity. Luteolin has been shown to bind to the ERs, to elicit an estrogenic response using an ERE-containing promoter reporter construct, to induce breast cancer cell proliferation, and to antagonize E_2_-induced breast cancer cell proliferation, however, unlike F3, luteolin does not display a significant preference for ERβ signaling [29,30,66,67].

Since none of the major compounds in F3 independently appeared to be responsible for the robust ERβ agonism observed, to evaluate their combined effect we re-engineered F3 using the major quantifiable compounds (Table 2). However, neither the re-engineered F3 (F3R), nor the individual compounds present in F3R elicited any ERβ agonism. Thus, two possibilities may explain the robust ERβ agonism of F3: Firstly, the unidentified major compounds in F3 (Fig F in S4 Fig, approximately 7.4, 20.0, and 20.5 minutes) may be the elusive ERβ agonist(s), or secondly, the minor compounds in F3 (S1 Table) may individually, although unlikely as they are present in very low concentrations, or collectively contribute to the robust activity of F3.

In the field of nutraceutical development, the idea of intelligent mixtures in the investigation of activity, induced by a mixture of compounds, has become increasingly popular [48,68–72]. A mixture would allow for synergistic, additive or antagonistic effects to contribute to the desired estrogenic traits in that the combination of a large number of weakly active compounds may exert an effect without the necessity of the presence of one major highly active compound. For example, it has recently been demonstrated by Bartoszewski *et al*. [73] that even though hesperidin has been shown to elicit cell growth arrest and apoptosis in various cancer cell lines including pancreatic cancer cells, colon cells, and breast cancer cells [74–77], the presence of mangiferin results in an additive apoptotic effect. Furthermore, Kumar *et al*. investigated the synergistic effects of three phytoestrogens (genistein, quercetin and biochanin A) and found that these phytoestrogens in combination had a more potent inhibitory effect on androgen-responsive prostate cancer cell growth *in vitro* [21]. Synergism may not only be applicable to one target, such as the ER, but could also involve multiple targets. In fact, it has been suggested that in most botanical drugs multi target effects may actually predominate over other synergistic mechanism [78]. For example, it has been shown that a polyphenol-based matrix, due to the presence of specific polyphenols, can result in an increase [79] in the absorption of other polyphenols.

In summary, the current study illustrated that it is unlikely that one compound is responsible for the desired estrogenic attributes displayed by SM6Met, but rather that multiple compounds contribute to the activity of the extract, either individually (additive), or by eliciting a synergistic or antagonistic effect in the presence of other SM6Met compounds. Thus, the traditional paradigm in drug discovery of dividing a mixture, such as SM6Met, in the hope of discovering a single highly active compound influencing an individual target, such as the ER, may not be the optimal approach towards retaining the complex desirable estrogenic attributes of a *Cyclopia* nutraceutical extract. The concept of intelligent mixtures may therefore be more relevant to not only the robust ERβ agonism of F3, but also to the NPF, which displayed all three of the pursued desirable estrogenic attributes.

Thus the current study could serve as a stepping stone in the possible future development of not only a uniquely South African honeybush-derived oral phytoestrogenic nutraceutical but may also be applicable to other studies of complex mixtures displaying desirable attributes that are complex in encompassing more than one molecular target.

## Supporting Information

S1 Fig SM6Met production and fractionation scheme. M6 *C*.*subternata* harvesting was subjected to sequential methanol extraction according to Mfenyana *et al*. [1]. The resultant SM6Met was fractionated using liquid-liquid fractionation to yield the PF and the NPF, which was subsequently fractionated into F1, F2, and F3 using HPCCC. Experimental details are given in the Materials and Methods section. Yields are expressed as if 100 g SM6Met was used in fractionation. Values in brackets represent the actual mass used during the study.
(TIF)

S2 Fig Representative high performance counter-current chromatogram (HPCCC) of the NPF. Specific fractions were pooled to form F1 (22–40 min), F2 (41–52 min) and F3 (52–75 min).
(TIF)

S3 Fig Estrogen receptor characterization of the HEK293 and MCF-7 BUS cell lines. Western blot analysis reveals (A) the absence of ERα and ERβ in the HEK293 cell line and the (B) presence of ERα and ERβ in the MCF-7 BUS cell line. The + Control represents lysates from COS-1 cells transfected with the cognate receptor, while the—Control represents lysate from untransfected COS-1 cells. Western blot analysis was performed as previously described [2].
(TIF)

S4 Fig LC-MS/MS chromatograms of SM6Met and subsequent fractions obtained during activity-guided fractionation. (A) SM6Met, (B) the NPF, (C) the PF, (D) F1, (E) F2, and (F) F3. Chromatograms indicate base peak intensity (BPI) with the peak numbers corresponding to the numbering in S1 Table. The initial peak in all samples corresponds to ascorbic acid (not numbered), which was added to prevent oxidation. Peaks above the dotted lines were considered major peaks, whilst peaks below the dotted lines were considered minor peaks as indicated in S1 Table.
(TIF)

S1 Table LC-MS/MS data (S4 Fig) of compounds present in SM6Met and subsequent fractions obtained during activity-guided fractionation. (PDF)

S2 Table Purification table of estrogenic activity. (PDF)
