# Supplementary material for: Divide and Conquer May Not Be the Optimal Approach to Retain the Desirable Estrogenic Attributes of the Cyclopia Nutraceutical Extract, SM6Met
Source: PLoS One. 2015 Jul 24;10(7):e0132950. doi: 10.1371/journal.pone.0132950 (PMC4514865; doi:10.1371/journal.pone.0132950)
Supplement: S1 Table — (PDF) [file pone.0132950.s005.pdf]

S1Table. LC-MS/MS data (S4 Fig.) of compounds present in SM6Met and subsequent fractions obtained during activity-guided fractionation.

Nr	t_R[a]	λ_{max}	$[M-H]^-$	Fragment ions	Compound	Class	Notes[b]	SM6Met	PF	NPF	F1	F2	F3
1	2.7	293	569	479, 449, 317, 287*[c]	Iriflophenone-3-*C*-β-D-glucoside-4-*O*-β-D-glucoside	Benzophenone	ID	x[d]	**X**[e]	x	x		
2	4.1	259, 293	153	No fragments observed	Protocatechuic acid	Hydroxybenzoic acid	ID	**X**		**X**			**X**
3	5.6	286	611	491, 431, 401, 371*	(*S*)-Eriodictyol-di-*C*-hexoside	Flavanone	[3]	x	x	x	x		
4	5.7	286	611	491, 431, 401, 371*	(*R*)-Eriodictyol-di-*C*-hexoside	Flavanone	[3]	x	x	x	x		
5	5.8	294	407	317, 287*, 257, 245, 215, 201, 193, 165, 125	Iriflophenone-3-*C*-β-D-glucoside	Benzophenone	ID	**X**	x	**X**		**X**	x
6	7.3	285	595	475, 415, 385*, 355	Naringenin-di-*C*-hexoside	Flavanone	[3]	x	x	x	x		
7	8.4	239, 257, 317, 366	421	331, 301*, 271, 259	Mangiferin	Xanthones	ID	**X**	x	**X**			**X**
8	8.8	240, 255, 316, 365	421	331, 301*, 271, 259	Isomangiferin	Xanthones	ID	**X**	x	**X**			**X**
9	9.0	231, 270, 330	593	503, 473*, 383, 353	Vicenin-2 (apigenin-6,8-di-*C*-β-D-glucoside)	Flavone	ID	x	x	x	**X**	x	
10	10.1	281	449	287, 151*, 135	Eriodictyol-*O*-glucoside	Flavanone	[3]	x		x			x
11	11.2	285	613	475, 433, 403, 373*, 361, 331, 209	3-Hydroxyphloretin-3',5'-di-*C*-hexoside	Dihydrochalcone	[3]	**X**	**X**	**X**	**X**	x	x
12	11.3	309	163	No fragments observed	*p*-Coumaric acid	Hydroxycinnamic acid	ID	x	x	**X**			**X**
13	12.0	283	595	287, 151*	Eriocitrin (eriodictyol-7-*O*-rutinoside)	Flavanone	ID	**X**	x	**X**	**X**	**X**	
14	13.0	265, 348	593	285	Scolymoside (luteolin-7-*O*-rutinoside)	Flavone	ID	**X**	**X**	**X**		x	**X**
15	13.3	284	597	459, 417, 387, 357*, 345, 315	Phloretin-3',5'-di-*C*-β-D-glucoside	Dihydrochalcone	ID	**X**	**X**	**X**	**X**	x	x
16	13.6	254, 265, 348	447	285	Luteolin-*O*-hexoside	Flavone	NC	x		x			x
17	14.3	282	579	271*, 151	Naringenin-*O*-dihexoside	Flavanone	[3]	x	x	x			x
18	15.0	266, 337	577	269	Isorhoifolin (apigenin-7-*O*-rutinoside)	Flavone	NC	x	x	x			x
19	15.5	283	609	301	Hesperidin (hesperetin-7-*O*-rutinoside)	Flavanone	ID	**X**	**X**	**X**	x	**X**	x
20	15.8	252, 265, 346	607	299*, 284	Chrysoeriol-*O*-deoxyhexose-*O*-hexoside	Flavone	NC	x	x	x			x
21	18.0	259, 375	609	301	Quercetin-*O*-deoxyhexose-*O*-hexoside	Flavonol	NC	x				x	
22	21.0	252, 265, 348	285	No fragments observed	Luteolin	Flavone	ID	x		x			x

[a] indicates the polyphenol retention time
[b] MS data from literature used to tentatively identify compounds
[c] * indicates the major daughter ion
[d] Minor polyphenols in extract are represented by “x”
[e] Major polyphenols in extract are represented by “**X**’’
ID refers to the fact that the compound was identified by means of an authentic reference standard
NC refers to the presence of a compound not identified before and further structure elucidation is required
