# Supplementary material for: Divide and Conquer May Not Be the Optimal Approach to Retain the Desirable Estrogenic Attributes of the Cyclopia Nutraceutical Extract, SM6Met
Source: PLoS One. 2015 Jul 24;10(7):e0132950. doi: 10.1371/journal.pone.0132950 (PMC4514865; doi:10.1371/journal.pone.0132950)
Supplement: S2 Table — (PDF) [file pone.0132950.s006.pdf]

S2 Table. **Purification table of estrogenic activity.**

	ERα				ERβ			Proliferation		
	**Agonist activity**		**Antagonist activity**		**Agonist activity**		**Antagonist activity**	**Agonist activity**	**Antagonist activity**	
	**Fold[a]**	**PuF[b]**	**% decrease[c]**	**PuF**	**Fold**	**PuF**	**% decrease**	**Fold**	**% decrease**	**PuF**
**SM6Met**	1.8 [#]	1.0	30% [##]	1.0	2.2 [###]	1.0	—	—	18% [##]	1.0
**PF**	—		36% [###]	1.2	—	—	—	—	17% [##]	0.94
**NPF**	—		41% [###]	1.4	2.3 [###]	1.1	—	—	17% [##]	0.94
**F1**	—		52% [###]	1.7	—	—	46% [###]	—	—	—
**F2**	—		54% [###]	1.8	—	—	—	—	16% [###]	0.90
**F3**	1.6 [###]	0.9	—		4.5 [##]	2.1	—	1.4 [ns]	—	—

[a] Fold-induction relative to solvent (Figs. 2 and 3)

[b] PuF refers to purification factor (activity of compound in fraction/ activity of compound in SM6Met)

[c] Percentage decrease in induction relative to 10^{-11} M E_2 set as 100% for promoter reporter studies and 10^{-9} M E_2 set as 100% for proliferation studies (Figs. 2&3)

— refers to no activity: for agonist mode this implies no statistical difference from solvent, while for antagonist mode it implies no statistical difference from 10^{-11} M E_2 for promoter reporter studies and 10^{-9} M E_2 for proliferation studies.

Statistical analysis was done using One-way ANOVA with Dunnett's post-test comparing all values to E_2 ([#], $P<0.05$; [##], $P<0.01$; [###], $P<0.001$).
